# COVID-19 targets the right lung

**DOI:** 10.1186/s13054-020-03033-y

**Published:** 2020-06-15

**Authors:** Jingwen Li, Xiaxia Yu, Shaoping Hu, Zhicheng Lin, Nian Xiong, Yi Gao

**Affiliations:** 1 Department of Neurology, Union Hospital, Tongji Medical College, Huazhong University of Science and Technology, Wuhan, Hubei China; 2 School of Biomedical Engineering, Health Science Center, Shenzhen University, Shenzhen, Guangdong China; 3 Department of Radiology, Wuhan Red Cross Hospital, Wuhan, Hubei China; 4 Harvard Medical School, Belmont, MA 02478 USA; 5 Medical Treatment Expert Group for Covid-19, Wuhan Red Cross Hospital, Wuhan, Hubei China

Previous imaging studies of COVID-19 suggested that bilateral lungs be affected [1]. In this study, we noticed a side-preference of lung lesions in COVID-19. The lesions on the right lung were significantly larger and developed faster than those on the left. Moreover, the level of right-over-left preference of lung injury was significantly correlated with the potential need for intensive care and inpatient mortality.

Pulmonary lesions were imaged by a total of 253 high resolution computed tomographic (CT) chest scans of 103 COVID-19 patients at Wuhan Red Cross Hospital. Of the 103 patients with a median (interquartile range (IQR)) age of 63 (47–70) years, 57 were males, 41 were in the intensive care unit (ICU), and 64 were deceased during hospitalization. At the time of the study, only 1 patient was still in the ICU. The median (IQR) length of known hospital stay was 16 (10–24) days.

Two independent assessments of the images were conducted. First, one physician visually evaluated the level of injury on either side of the lung and provided percentage-based semiquantitative scores on the injury of the lung for either side (0: no, 1: < 5%, 2: 6–25%, 3: 26–50%, 4: 51–75%, 5: > 75% injury). Second, an automatic deep learning-based algorithm [2] extracted the three-dimensional (3D) features of all lesions that were validated by physicians. Then, quantitative volumetric analysis and comparisons were carried out for right-versus left-side lesions’ volumes and their growth speed.

As a result, 70% (31/103) CT scans showed that the lesion volume of the right lung was larger than that of the left lung. Figure 1 shows a typical case in panels a and b. In addition to the first-time CT of each patient, we studied 253 longitudinal CT images of 103 patients (2–3 time points/patient).

**Fig. 1:**
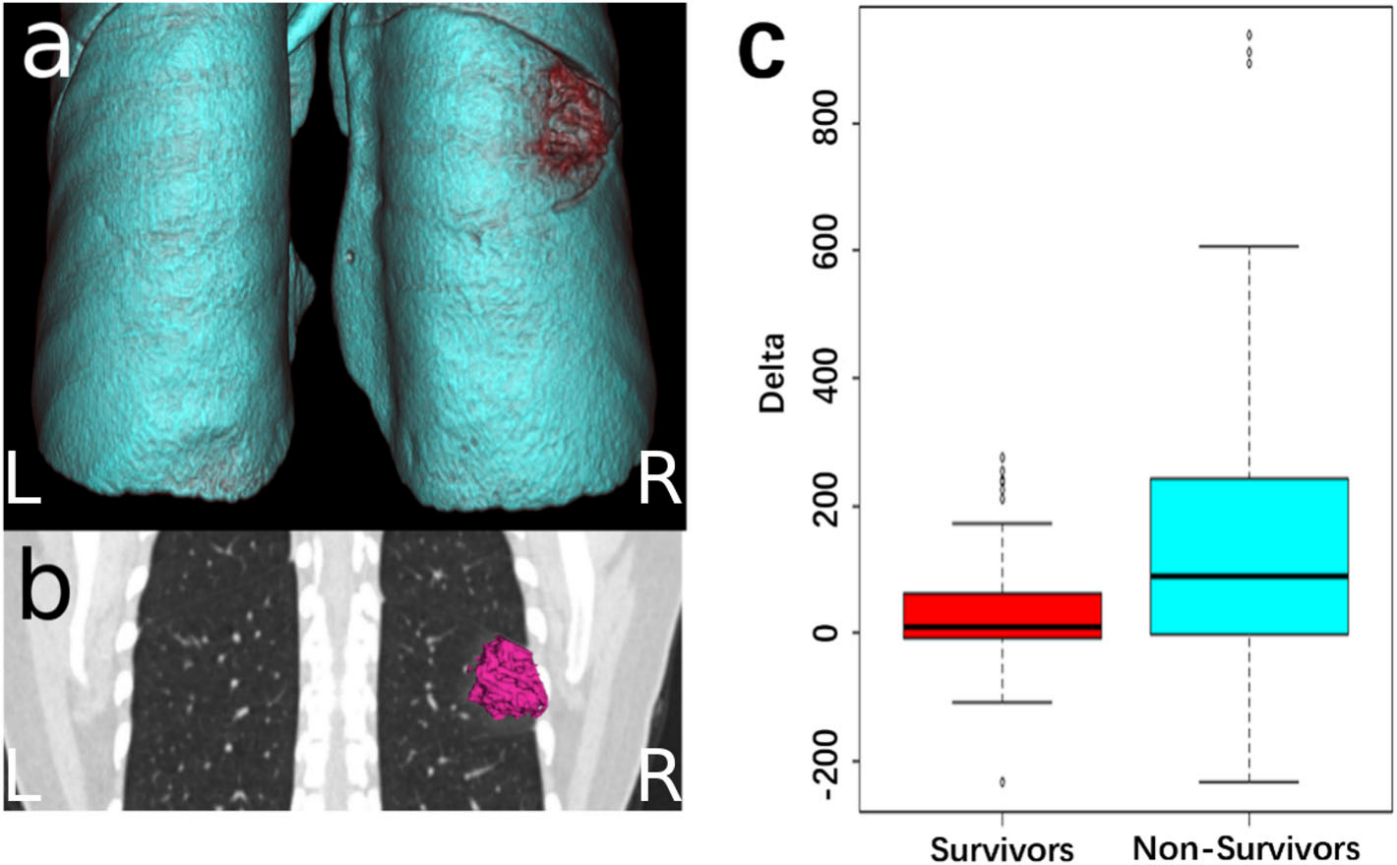
**a** 3D renders the highlights of a lesion on the right lung (view from posterior). **b** 3D extraction of the lesions. **c** Comparison of Δ in non-surviviors and survivors (one-sided *t* test, *P* < 0.0001)

In order to facilitate the discussion below, here we denoted Delta (Δ) as the difference of the volume of the lesion on the right lung and that of the lesion on the left lung. A potential association between the age and Δ was also analyzed. The results showed that the older the patient, the larger the Δ (*χ*^2^ test for linear terms, *F* = 11.9466, *P* < 0.0001) (Fig. 2a). Moreover, the non-survivors had bigger Δ than the survivors (one-sided *t* test, *P* < 0.0001) (as shown in Fig. 1c). Smooth curves were fitted for Δ and a log risk ratio was used for death. These results indicated the lowest risk ratio when the Δ was around 0, and the differences between the left and right lungs were minimal. The risk for death increased with larger Δ (*P* = 0.013, Fig. 2b). In order to understand which side was correlated with a severe form of the disease, Fisher’s extract test was used to compare the fatality risk of patients with smaller lesions on the left side to those with larger lesions on the left. The results suggested that patients with large lesions on the right lung be at a high mortality risk during hospitalization (OR = 2.662, *P* value = 0.0252 (Fig. 2c, d).

**Fig. 2:**
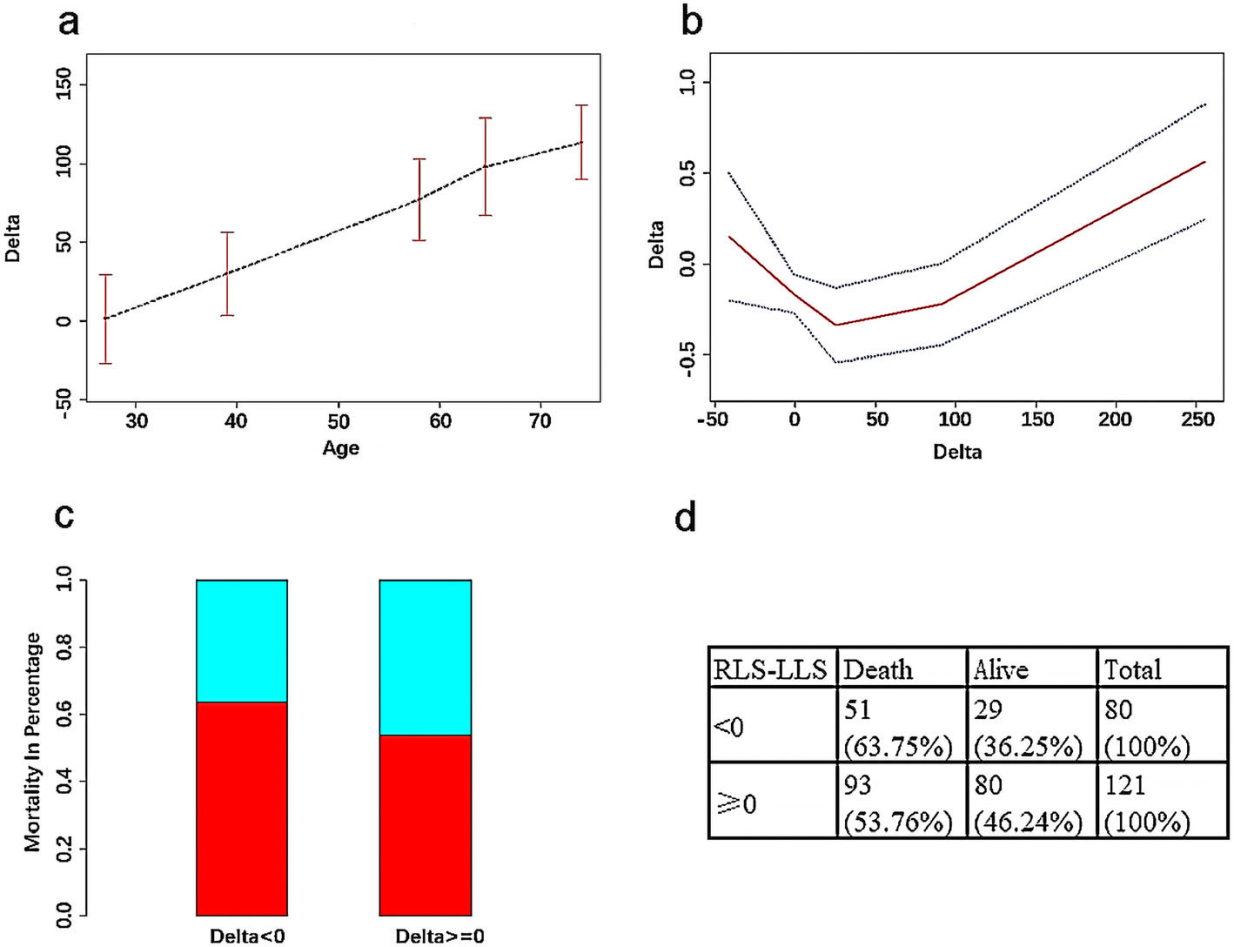
Statistical analysis. **a** Correlation between age and delta. Age was categorized into five groups. **b** Association between Δ and relative death ratio. **c** Comparison of mortality in patients with Δ < 0 and Δ ≥ 0. Mortality rate of non-survivors was shown in cyan; mortality rate of survivors was shown in red. **d** Contingency table for Δ and mortality

Different diseases have different origins of lesions. For instance, foreign bodies are likely to be aspirated in the right bronchus because of the short, wide, and straight path [3]. Tuberculosis prefers the right upper lobes [4], which might be attributed to the oxygen distribution ratio. In COVID-19, the right side-preference was consistent with a reported autopsy result that the right lung was subject to hemorrhage [5]. These results draw care attention to the right lung in this novel pneumonia.

## Data Availability

The datasets used and/or analyzed during the current study are available from the corresponding author on reasonable request.
